# Enhancer-derived RNA: A Primer

**DOI:** 10.1016/j.gpb.2016.12.006

**Published:** 2017-05-19

**Authors:** Feng Liu

**Affiliations:** National Research Center for Translational Medicine, Ruijin Hospital, Shanghai Jiao Tong University School of Medicine, Shanghai 200025, China

**Keywords:** eRNA, Enhancer, Next-generation sequencing, Non-coding RNA, Gene regulation

## Abstract

Enhancer-derived RNAs (**eRNAs**) are a group of RNAs transcribed by RNA polymerase II from the domain of transcription **enhancers**, a major type of *cis*-regulatory elements in the genome. The correlation between eRNA production and enhancer activity has stimulated studies on the potential role of eRNAs in transcriptional regulation. Additionally, eRNA has also served as a marker for global identification of enhancers. Here I review the brief history and fascinating properties of eRNAs.

## Introduction

The explosive growth of next-generation sequencing (NGS) technologies has revolutionized the studies to comprehensively interrogate the transcriptionally active fraction of the genome. NGS has not only made it possible to exhaustively catalog the transcripts from coding sequences (*i.e.*, genes), but also has facilitated the discovery of many RNA species that do not serve as template for protein synthesis [1]. Over the past decade, many studies have shown that several classes of non-coding RNAs (ncRNAs), including microRNAs (miRNAs) and long ncRNAs (lncRNAs), play diverse biological roles such as post-transcriptional regulation of mRNA stability and epigenetic control of chromatin activity [2], [3]. These studies have greatly enriched our understanding of the composition and functional operation of the genome.

**Figure 1:**
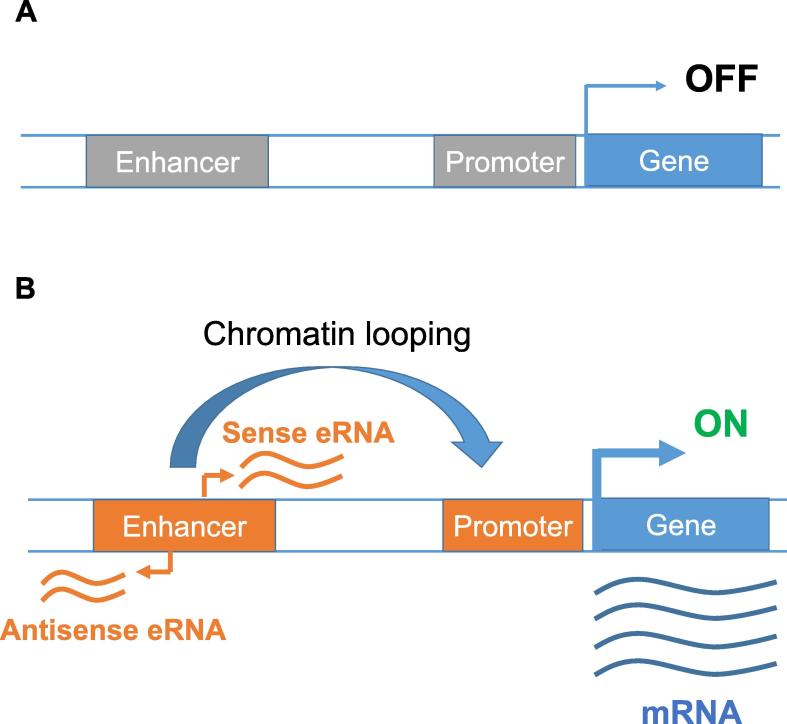
**eRNA and transcriptional activation** A typical gene is associated with two types of *cis*-regulatory elements: one proximal (the promoter) and the other distal (the enhancer) to the transcription start site of the gene. Except for house-keeping genes, a gene’s transcriptional activity is usually “off” when its enhancer(s) is inactive (**A**). However, when an enhancer is activated by transcription factors, it can loop toward the promoter and turn “on” the transcription of the gene (**B**). Previously, both enhancers and promoters were classified as non-coding elements, yet recent studies indicate that active enhancers are bi-directionally transcribed to eRNAs. eRNA, enhancer-derived RNA.

Of the ncRNA family, a latecomer and somewhat unconventional member is the enhancer-derived RNAs (eRNAs), which are transcribed at the loci of enhancers [4] (Figure 1). Like other *cis*-regulatory elements (CREs) such as promoters and insulators, enhancers contain binding sites—DNA sequence motifs varying in length between 6 bp and 20 bp—of various transcription factors (TFs) [5]. Through binding with several TFs, each enhancer acts as the nucleating site to form large multi-protein complexes to activate transcription [4]. Recent studies suggest that the human genome harbors millions of enhancers that can be activated at different developmental stages and in various tissues and cell types [1].

While enhancers and TFs are the primary players of gene regulation, other factors capable of chemically modifying DNA and histones are gradually being incorporated into the overall scheme of gene regulation, many of which modulate the accessibility of chromatin to allow for direct contact between TFs and enhancers [6]. Thus far, a number of enzymes with the ability to add or remove methyl-, acetyl- and phosphor-groups to DNA and histone tails are being deemed as critical gene regulators [7]. It is generally thought that involvement of these factors impinges additional layers of regulation, and thus improves the specificity of gene expression patterns [8]. Nevertheless, how a multitude of factors with varying degrees of overlapping functions are integrated into singular transcription outputs still presents a conceptual block for molecular biologists who are interested in understanding the nuisances of gene regulation [4]. It is in this ever increasingly complex picture that the eRNAs unexpectedly showed up, raising the question of whether they are a missing piece of the puzzle for the mechanism of gene regulation [9], [10].

## What is eRNA?

Although transcription from active enhancers was reported in the early 1990 s [11], [12], eRNAs were brought to spotlight by two reports in 2010 [13], [14]. In both studies, high-throughput sequencing was used to characterize stimulus-dependent enhancers, unexpectedly revealing broad RNA polymerase II (RNAPII)-mediated transcription of bi-directional eRNAs, typically 0.5–2 kb in length [14] (Figure 1B). Moreover, the expression levels of eRNAs correlate with the *cis*-regulatory activity of their template enhancers—that is, context-dependent, enhancer-stimulated mRNA synthesis of nearby genes—indicating an intimate association between enhancer function and eRNA production [13], [14]. Pervasive RNA synthesis from enhancers was soon confirmed in the majority of human cell types and tissues through systematic characterization of the functional elements in the genome by the Functional Annotation of the Mammalian Genome (FANTOM) consortium [15] and the Encyclopedia of DNA Elements (ENCODE) consortium [1].

## How are eRNAs detected?

Because the abundance of eRNAs is 19–34-fold lower than that of gene transcripts [15], relative higher coverage NGS, as compared to regular RNA-sequencing (RNA-seq), is required to accurately determine the places where enhancers are transcribed. In one of the first reports of genome-side eRNA synthesis, transcripts from enhancers were detected in sequencing experiments using total RNAs but not polyadenylated (polyA+) RNAs only [14], although later studies suggested that some eRNAs are polyadenylated, similar to other lncRNAs [16].

eRNAs are not as stable as mRNAs [17]. Therefore, a more robust method to identify eRNAs requires the capture of pioneering rounds of transcription using techniques such as global nuclear run-on sequencing (GRO-seq) [18] and precision nuclear run-on sequencing (PRO-seq) [19], [20]. Additionally, a sensitive way to detect eRNAs is cap analysis of gene expression (CAGE) sequencing [21]. This method was used by the FANTOM consortium to profile the transcriptomes of a large panel of human tissues and cell types, from which 43,011 enhancer elements were shown to be transcribed to eRNAs [22].

To assist with the enrichment of eRNAs, chromatin immunoprecipitation (ChIP) using antibodies against histone variants (*e.g.*, H2AZ) or modifications (*e.g.*, H3K27ac and H3K4me1) can be applied in eRNA detection experiments [23]. Another method to enhance eRNA detection, named BruUV-seq, uses UV light to introduce transcription-blocking DNA lesions, followed by bromouridine-labeling and deep sequencing of nascent RNAs [24].

Furthermore, eRNAs, like other RNA transcripts, can be visualized by *in situ* hybridization, using complementary RNA probes labeled with biotin or fluorescein [25]. When two or more RNA probes are used to simultaneously detect the eRNA and nearby protein-coding transcript(s), the dynamic relationship between an enhancer’s transcriptional activity and its gene-regulating function can also be investigated [25].

While these methods all provide objective ways to characterize eRNAs, enhancers are active only at selected tissues and cells [6]. Therefore, transcription from active enhancers, in principle, is expected to occur only in a spatially and temporally restricted manner. Consequently, analysis of the dynamic expression patterns of eRNAs is most fruitful when performed in the cellular context where their enhancers are functionally active.

## The function of eRNAs

Despite strong correlation between eRNA synthesis and enhancer activity, it remains unclear whether there is a mechanistic link between these two. On the one hand, it has been suggested that eRNAs may function as transcription activators [9], [10]. On the other hand, eRNAs may just be a result of spurious transcriptional activities as RNAPII is recruited to the neighborhood of enhancers [9]. Note that the latter is not a simple null hypothesis in light of the finding by the ENCODE consortium that approximately 80% of the human genome is capable of being transcribed, yet less than 50% of the genome are known to contain CREs and coding sequences [1]. This suggests that transcriptional activity can occur in about 30% of the genome that does not encode genes or CREs.

In support of a role of eRNAs in augmenting enhancer activity, studies using RNA interference (RNAi) to deplete several eRNAs in human cells found evidence for a causal role of eRNAs in transcriptional activation [18], [26], [27], [28], [29]. Furthermore, other studies suggested that eRNAs can interact with the Mediator and the cohesion complexes to establish chromatin looping, which is essential for the interaction between enhancers and promoters [27], [30]. One caveat of these earlier studies, however, is that the functional importance of eRNAs primarily derives from experiments using traditional RNAi techniques to knock down eRNAs, yet the majority of eRNAs are located within the nucleus, where RNAi does not work as effective as it does in the cytoplasm [31].

A more rigorous approach to investigate the function of the eRNA is to interfere with its synthesis by inserting a polyadenylation cassette near its transcription start site, which triggers premature transcription termination. In a recent study, this approach was applied on the locus of an enhancer regulating the expression of *cdkn1b* [32]. Notably, while transcription from this enhancer is reduced by >90%, transcription of its target gene, *cdkn1b*, is largely intact [32]. This suggests that the eRNA from this enhancer is an inert by-product during gene transcription [32]. Nevertheless, since using this approach to truncate eRNA production has not been carried out on a large scale, it remains unclear how many eRNAs are likewise dispensable for enhancer activities.

## eRNAs are markers of enhancers

Since the completion of the Human Genome Project, a major focus of the scientific community is to develop effective means to precisely map the estimated millions of CREs orchestrating distinct gene expression patterns of each cell type and developmental stage [1]. Currently, global enhancer mapping methodologies primarily rely on three parameters correlated with enhancer activation: (1) TF (or cofactor) binding; (2) distinct histone modifications at enhancer loci; and (3) accessible “open” chromatin [8]. Here, it is noteworthy that “open” chromatin is the state of the chromatin during enhancer activation, which is not necessarily the cause or the result of it. Under the same rubric, regardless of whether eRNAs contribute to the function of enhancers, high-throughput eRNA detection can be exploited to globally map enhancers.

Indeed, thousands of enhancers have been identified through analyzing the transcripts from non-coding sequences, which exhibit excellent overlap with the enhancer maps generated by histone mark ChIP-seq [22]. The strong correlation between an enhancer’s activity and the expression levels of eRNAs at a chromatin domain has also enabled studies to use eRNAs as a surrogate to investigate the effects of interrupting transcription regulators by short hairpins or small molecules (*e.g.*, JQ1) [33], [34]. In these studies, side-by-side comparison of eRNAs and protein-coding transcripts provides a straightforward way to establish the mechanistic link between an enhancer and its nearby gene(s).

As the cost of high-throughput sequencing rapidly decreases, protocols allowing for accurate determination of eRNAs become more feasible over time. Given that RNA-seq is now routinely used in genomic characterization of cells and tissues, using eRNAs to predict enhancer activity can obviate the need for additional experiments to identify enhancers. This is an advantage over other enhancer mapping techniques such as ChIP-seq, DNase-seq, and assay for transposase-accessible chromatin (ATAC)-seq, all of which use protocols different from RNA-seq. Thus, eRNA analysis may be especially useful in situations where specimen supply is limited (*e.g.*, in clinical settings) [21]. For example, He et al. [35] recently examined the occurrence of an ultra rare single-nucleotide mutation in chromosome 4q32 (4q32A > C) in a large pedigree displaying non-medullary thyroid carcinoma (NMTC). Interestingly, the expression level of eRNAs from this region is greatly down-regulated in NMTC tumors, suggesting that, as a marker of enhancer activity, eRNA measurement can be used to validate disease-causal mutations. In another study, androgen receptor-regulated eRNAs (AR-eRNAs) were used to monitor the response of castration-resistant prostate cancer (CRPC) to enzalutamide, a second-generation anti-androgen compound, leading to the discovery of a number of gene loci that contribute to enzalutamide-resistant growth of CRPC [36].

## The future of eRNA studies

It is fair to say that there still lacks a consensus on the biological function of eRNAs, especially regarding their relationship with enhancer activity. This lack of clarity is not entirely unexpected, considering that eRNAs became known just six years ago [13], [14]. It is anticipated that more mechanistic studies will be performed to reach a mature conclusion about this relatively young member of the ncRNA family. Along this direction, one area that warrants attention is the development of new tools to manipulate eRNAs in the cell, which is critical for both functional studies and for exploiting eRNA as therapeutic targets [37]. One potentially useful method is the locked nucleic acid (LNA) technology that can target nuclear-located eRNAs with high efficiency, which may be more useful than traditional RNAi techniques [31]. Moreover, recently developed clustered regularly interspaced short palindromic repeats (CRISPR)/CRISPR-associated protein 9 (Cas9) technology has made it feasible to perform large-scale genome editing experiments [38]. A systematic effort to manipulate eRNA synthesis *in situ*—for example, by targeting insertion of transcription premature termination signals in enhancer loci—will lead to a comprehensive view of the role of eRNAs in the *cis*-regulatory activity of enhancers.

Thus far, several studies have implicated a role of eRNAs in engaging the Mediator and the cohesion complexes, while the latter two mediate chromatin looping [27], [30], [39]. In the future, a thorough analysis of eRNA-binding proteins may help illuminate where and how eRNAs fit into the complex network of interactions during transcriptional regulation. This likely requires the development of robust and scalable methods to systematically identify eRNA-interacting proteins [40].

Finally, the growing need to decipher the epigenome in both normal and disease tissues is expected to demand ever more sensitive and comprehensive methodologies to characterize enhancers in various cellular contexts. Notably, the past decade has seen the documentation of a plethora of disease-associated genetic variants (*e.g.*, single nucleotide polymorphism and copy number variation) and mutations that appear to be enriched in putative enhancer elements [41]. As eRNAs are a useful marker of active enhancers, targeted sequencing and bioinformatics analysis of eRNAs may accelerate functional annotation of these genetic variants and mutations in human diseases [21].

## Competing interests

The author declares no competing interests.
